# Mle046 Is a Marine Mesophilic MHETase-Like Enzyme

**DOI:** 10.3389/fmicb.2021.693985

**Published:** 2021-07-26

**Authors:** Ingrid E. Meyer-Cifuentes, Başak Öztürk

**Affiliations:** Junior Research Group Microbial Biotechnology, Leibniz Institute DSMZ-German Collection of Microorganisms and Cell Cultures, Braunschweig, Germany

**Keywords:** biodegradable plastics, esterases, marine biotechnology, enzymology, marine bacteria

## Abstract

Accumulation of plastics in the oceans presents a major threat to diverse ecosystems. The introduction of biodegradable plastics into the market aims to alleviate the ecological burden caused by recalcitrant plastics. Poly (butylene adipate-co-terephthalate) (PBAT) is a biodegradable commercial plastic that can be biodegraded similarly to polyethylene terephthalate (PET) by PETase-like enzymes and MHETases. The role of MHETases is to hydrolyze the intermediate degradation product of PET, mono-2-hydroxyethyl terephthalate (MHET) to its monomers. We recently identified a homolog of the MHETase of the PET-degrading bacterium *Ideonella sakaiensis*, Mle046, from a marine microbial consortium. In this consortium, Mle046 was highly expressed when a PBAT-based blend film (PF) was supplied as the sole carbon source. In this study, we recombinantly expressed and biochemically characterized Mle046 under different conditions. Mle046 degrades MHET but also 4-(4-hydroxybutoxycarbonyl) benzoic acid (Bte), the intermediate of PF degradation. Mle046 is a mesophilic enzyme adapted to marine conditions, which rapidly degrades MHET to terephthalate and ethylene glycol at temperatures between 20 and 40°C. Mle046 degradation rates were similar for Bte and MHET. Despite its mesophilic tendency, Mle046 retains a considerable amount of activity at temperatures ranging from 10 to 60°C. In addition, Mle046 is active at a range of pH values from 6.5 to 9. These characteristics make Mle046 a promising candidate for biotechnological applications related to plastic recycling.

## Introduction

**FIGURE 1:**
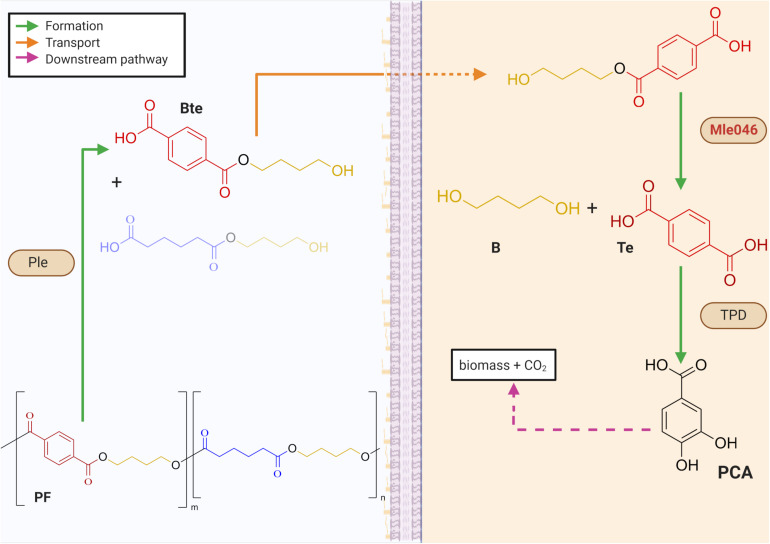
Mechanisms for the biodegradation of a PBAT-based blend film (PF) within a biofilm. Periplasmatic α/β hydrolases (Ples) cleave the PF to produce oligomers of 1,4-butanediol (B) with either terephthalic acid (Te) or adipic acid. Mles (e.g., Mle046 shown in red) hydrolyzes inside the cell a mixture of terephthalate-butanediol monoester (Bte) that yields Te and B. Te is further degraded by a Te degradation cluster (TPD). Downstream degrading enzymes lead to the formation of protocatechuate (PCA) which can be further degraded to biomass and CO_2_. Green arrows indicate the formation of intermediates after enzymatic catalysis; orange arrows show the transport of intermediates to the cell; pink-dashed arrows indicate downstream degradation pathways (created with Biorender.com).

Biodegradable plastics were introduced in the 1980s into the market as an ecologically friendlier substitute to non-biodegradable plastics (Chen, 2009). Most of these biodegradable plastics contain ester bonds that are prone to enzymatic hydrolysis. One of these biodegradable plastics is the copolymer poly (butylene adipate-co-terephthalate (PBAT), composed of the monomers terephthalic acid (Te), adipic acid, and 1, 4-butanediol (B). The combination of aliphatic and aromatic units in PBAT offers better physical properties than aliphatic–aliphatic biodegradable plastics (Jian et al., 2020). Their chemical structure gives them flexibility and strength similar to low-density polyethylene plastic (Jian et al., 2020). In contrast to polyethylene, however, PBAT is susceptible to microbial degradation in compost and soils (Witt et al., 1999; Zumstein et al., 2018). PBAT biodegradability and high performance make it suitable for packing films. This makes them more economically attractive than other biodegradable plastics (Ferreira et al., 2019). As already mentioned, PBAT-based plastics can be biodegraded easily in compost by microorganisms (Witt et al., 1995, 1996, 2001). Their fate and biodegradability in aquatic natural environments, however, are still poorly understood. In a previous study, we revealed that a synergistic mechanism within a marine consortium was necessary to achieve complete mineralization of a PBAT-based polymer blend (PF) (Meyer-Cifuentes et al., 2020). In that study, several putative PETase-like (Ple) and MHETase-like (Mle) hydrolases as well as terephthalic acid degrading genes (TPD) were found in the metagenome. We proposed then the following mechanism for PF biodegradation: Ples hydrolyze the ester bonds of the polymeric PF, yielding the monoester 4-(4-hydroxybutoxycarbonyl) benzoic acid (Bte) and other oligomers. Bte can be further degraded by highly specific esterases, namely, Mles (Figure 1). In this respect, the suggested biodegradation mechanism for PF is similar to that of polyethylene terephthalate (PET) degradation by *Ideonella sakaiensis* (Yoshida et al., 2016). Degradation of PET by this bacterium is initiated by IsPETase to yield the monoester mono-2-hydroxyethyl terephthalate (MHET), the first intermediate of PET degradation. MHET is then further hydrolyzed by α/β-hydrolase, IsMHETase, to Te and ethylene glycol. *I. sakaiensis* can further degrade Te via a TPD cluster. Among the different Mles identified in our previous study (Meyer-Cifuentes et al., 2020), only Mle046, presumably produced by an Alphaproteobacterial strain, was consistently expressed and produced during PF degradation. This *mle046* is 100% identical to a homolog present in *Celeribacter manganoxidans* and its protein is 46.9% identical to IsMHETase. At the structural level, Mle046 contains a domain typical of feruloyl esterases compromising the canonical catalytic triad of these types of enzymes. The high expression in the presence of PF and its similarity to IsMHETase suggests that Mle046 is responsible for the PF degradation intermediate Bte and can also degrade the structurally similar PET degradation intermediate MHET.

In this study, we investigate the biochemical characteristics of a new MHETase-like enzyme, Mle046. Specifically, we demonstrate the ability of Mle046 to hydrolyze MHET, Bte under broad temperature and pH conditions. This novel MHETase-like enzyme could potentially be engineered in the near future to degrade plastics and produced plastic monomers in two-enzyme reactor systems as shown before (Barth et al., 2015; Knott et al., 2020).

## Materials and Methods

### Gene Synthesis

Codon-optimized gene *mle046* was synthesized by BioCat GmbH (Heidelberg, Germany) and cloned into a pColdII vector. The codon-optimized sequence can be found in Supplementary Table 1. The signal peptide (first 27 amino acids) of the original protein was detected with the SignalP software v. 5.0 (Almagro Armenteros et al., 2019) and was excluded from the synthetic construct.

### Expression of the Mle046 and Cell Lysis

The Mle046 was expressed following a protocol adapted from Palm et al. (2019). *Escherichia coli* Shuffle T7 cells (New England BioLabs, United States) were transformed with the pColdII plasmid containing the *mle046* insert. The transformants were plated on Lysogeny Broth (LB) agar plates with 50 mg/L of ampicillin and incubated at 37°C overnight. Pre-cultures were prepared by inoculating single colonies from the plates to liquid LB and incubated for 62 h at 20°C, 150 rpm. After this time, 1 mL of the pre-culture was inoculated to 200 mL of Terrific Broth containing 100 mg/L Carbenicillin (Sigma-Aldrich, United States) in 1 L baffled flasks. The cultures were incubated at 37°C at 150 rpm in a MaxQ^TM^ 4000 orbital shaker (Fischer Scientific, Germany), until an OD_600 *nm*_ of 0.8–1.0 was reached. Then 0.5 mM of IPTG was added to cultures pre-cooled on ice for 15 min. Cultures were incubated overnight at 15°C, 150 rpm. After incubation, the pellets were collected by centrifuging at 4°C, 5,514 × *g* for 10 min in a Heraeus^TM^ Multifuge^TM^ X3 (Fischer Scientific, Germany), and resuspended in His-Tag binding buffer (20 mM sodium phosphate and 0.5 M NaCl, pH 7.4) at 1:10 of the original culture volume. Cells were disrupted by incubation with FastBreak^TM^ Cell Lysis Reagent (Promega, United States) and 1 μL Pierce^TM^ Universal Nuclease for Cell Lysis (Thermo Fischer Scientific, United States) at 20°C for 30 min with constant shaking in a MaxQ^TM^ 4000 orbital shaker (Fischer Scientific, Germany). To separate and collect the soluble fraction, the lysates were centrifuged at 4°C, 15,000 × *g* for 30 min in a Heraeus^TM^ Multifuge^TM^ X3 (Fischer Scientific, Germany). The soluble fractions were filtered through a 0.2 μM filter.

### Identification and Purification of the Mle046

Ten microliters of soluble fractions of induced and non-induced cells were mixed with 10 μL of 2× sodium dodecyl sulphate–polyacrylamide gel electrophoresis (SDS-PAGE) Protein loading buffer and boiled for 10 min at 95°C in an Eppendorf Thermomixer^®^ C (Eppendorf, Germany). Insoluble fractions from the same experiments were resuspended in PBS and treated the same way as the soluble fractions as a control for denatured proteins or inclusion bodies. After cooling on ice, the samples were loaded into 12% SDS-PAGE gels and ran for 45 min at 25 V/cm in a Mini-PROTEAN^®^ Tetra Cell (Bio-Rad Laboratories, United States). The presence of Mle046 was detected by comparing induced to non-induced samples and to a protein size standard (Precision Plus Protein^TM^ Unstained Standards, Bio-Rad Laboratories, United States). As the protein was detected in the soluble fraction, insoluble fractions were not used for further experiments.

The soluble induced fractions were loaded into a 5 mL His-Trap^TM^ HP affinity Ni-Sepharose column (Cytiva, Germany) connected to an ÄKTA^TM^ Start Purification System (Cytiva, Germany). Proteins were bound and eluted from the column by using the predefined affinity purification protocol included in the UNICORN^®^ start 1.1 software (Cytiva, Germany). The purity of the fractions presenting a peak was assessed as described before by SDS-PAGE. The fractions were further concentrated on a Pierce^TM^ Protein Concentrator PES 10K MWCO (Thermo Fischer Scientific, United States). The affinity-purified fractions were further polished by size exclusion chromatography on a HiPrep^TM^ 16/60 Sephacryl^TM^ S-200 HR column (Cytiva, Germany) connected to an ÄKTA^TM^ Start Purification System (Cytiva, Germany) in a buffer consisting of 25 mM Tris–HCl and 200 mM NaCl, pH 7.5. Fractions containing the Mle046 protein were pooled and concentrated on a Pierce^TM^ Protein Concentrator PES 10K MWCO (Thermo Fischer Scientific, United States). The concentration of the purified protein was quantified by using the Qubit Protein Assay kit and measured on a Qubit 3.0 Fluorometer (Invitrogen, United States).

### Hydrolysis of MHET by Mle046

To assay the hydrolysis of MHET by Mle046, the activity of the enzyme was measured in triplicate as described before (Palm et al., 2019) with a few modifications. Mle046 was used at a final concentration of 0.002 μg/μL and incubated in the presence of different concentrations of MHET (300–2,700 μM) dissolved in 40 mM sodium phosphate buffer, pH 7.5. Incubations were carried out for 21 min at 30°C and shaking at 300 rpm in an Eppendorf Thermomixer^®^ C (Eppendorf, Germany). Every 3 min a sample was taken and immediately inactivated by adding an equal volume of methanol (Methanol Optigrade^®^ for HPLC, Promochem, LGC Standards GmbH, Germany). The samples were then centrifuged at 17,000 × *g* for 10 min in a Microstar 17R centrifuge (VWR, United States) and the supernatants were transferred to standard HPLC vials. Negative controls containing only the substrate and buffer were also included. MHET degradation was detected with a 1260 Infinity II LC System (Agilent Technologies, United States). Samples were separated through a Agilent Poroshell 120 HPH-C18 column (Agilent Technologies, United States) with a gradient of acetonitrile 99.9% HPLC grade (Fischer Scientific, United States) and 0.1% (v/v) formic acid (98–100% Suprapur^®^, Sigma-Aldrich, United States) in Milli-Q water. The flow rate was set at 0.2 mL/min. One microliter of the sample was injected. Acetonitrile was increased from 5 to 44% until minute 12 and then to 70% at minute 15 remaining constant for other 3 min. MHET was detected at 240 nm.

### Determination of Enzyme Kinetics

Mle046 rates were plotted against MHET concentrations, and the kinetic parameters were determined by using the GraphPad Prism v. 5. 01 software (GraphPad). Kinetics parameters such as maximum velocity (*Vmax*), Michaelis–Menten constant (*Km*), and the rate constant (*kcat*) were calculated with non-linear regression.

### Hydrolysis of Bte by Mle046 and Substrate Affinity

To assess Mle046 activity on the PF degradation intermediate, Bte, 0.002 μg/μL of the enzyme was incubated in 40 mM sodium phosphate buffer, pH 7.5 in the presence of 1,000 μM of Bte (Bte was synthetized as indicated in the Supplementary Material). Negative controls containing only the substrate and buffer were also included. Reactions and controls were prepared in triplicates and incubated at 30°C, 21 min, and shaking at 300 rpm in an Eppendorf Thermomixer^®^ C (Eppendorf, Germany). Every 3 min a sample was taken and immediately inactivated by adding an equal volume of methanol (Methanol Optigrade^®^ for HPLC, Promochem, LGC Standards GmbH, Germany). The samples were then centrifuged at 17,000 × *g* for 10 min in a Microstar 17R centrifuge (VWR, United States), and the supernatants were transferred to standard HPLC vials. MHET degradation was detected with a 1260 Infinity II LC System (Agilent Technologies, United States). Samples were separated through an Agilent Poroshell 120 HPH-C18 column (Agilent Technologies, United States) with a gradient of acetonitrile 99.9% HPLC grade (Fischer Scientific, United States) and 0.1% (v/v) formic acid (98–100% Suprapur^®^, Sigma-Aldrich, United States) in Milli-Q water. The flow rate was set at 0.2 mL/min. One microliter of the sample was injected. Acetonitrile was increased from 5 to 44% until minute 12 and then to 70% at minute 15 remaining constant for other 3 min. Bte and Te formation was detected at 240 nm.

Additionally, we tested substrate range of Mle046 by incubating the enzyme in the presence of a mixture of Bte and BHET (bis 2-hydroxyethyl terephthalate, the precursor to MHET during PET degradation), only with Bte or only with BHET. For this, 1 μL Mle046 (0.005 μg/μL) was incubated in 40 mM sodium phosphate buffer, pH 7.5 with the following substrates: 800 μM of BHET, 800 μM of Bte, and a mixture of 800 μM of BHET and 800 μM Bte. Negative controls containing only the substrate and buffer were also included. Reactions and controls were prepared in triplicates and incubated at 20°C for 30 min in an Eppendorf ThermoMixer^®^ C (Eppendorf, Germany). Three sampling points were taken at 0, 15, and 30 min. The samples were immediately inactivated after the addition of acidic (pH 2.5) 160 mM sodium phosphate buffer (Palm et al., 2019) and heating at 80°C for 10 min. Bte and BHET were further detected with a 1260 Infinity II LC System (Agilent Technologies, United States). Samples were separated through an Agilent Poroshell 120 HPH-C18 column (Agilent Technologies, United States). The mobile phase consisted of a gradient of acetonitrile 99.9% HPLC grade (Fischer Scientific, United States) and 0.1% (v/v) of formic acid (98–100% Suprapur^®^, Sigma-Aldrich, United States) in Milli-Q water. The flow rate was set at 0.2 mL/min. One microliter of the sample was injected. Acetonitrile was increased from 5 to 44% until minute 12 and then to 70% at minute 15 remaining constant for other 3 min. BHET and Bte were detected at 240 nm.

### Determination of Optimum pH and Temperature

We tested Mle046 activity at different temperatures and pH values by measuring the formation of Te after degradation of MHET at different pH and temperatures. For the reaction, Mle046 (0.002 μg/μL) was incubated in 100 mM sodium phosphate buffer pH 7.5 with 800 μM MHET. Negative controls containing only the substrate and buffer were also included. Reactions and controls were prepared in triplicates. Incubations were performed in a Veriti^TM^ 96-Well Thermal cycler (Applied Biosystems, United States) between 5 and 10–60°C with a 10°C increments. After 20 min, the reactions were immediately inactivated by adding an equal volume of methanol (Methanol Optigrade^®^ for HPLC, Promochem, LGC Standards GmbH, Germany). The samples were then centrifuged at 17,000 × *g* for 10 min in a Microstar 17R centrifuge (VWR, United States), and the supernatants were transferred to standard HPLC vials. To test the effect of different pH values on Mle046 activity, we incubated the enzyme with 800 μM MHET as the substrate in 100 mM sodium phosphate buffer. The pH of the buffer was adjusted between 5 and 10 with 1 pH unit increments. Negative controls containing only the substrate and buffer were also included. Reactions and controls were prepared in triplicates and incubated at 20°C in a Veriti^TM^ 96-Well Thermal cycler (Applied Biosystems, United States). After 20 min, the reactions were immediately inactivated by adding an equal volume of methanol (Methanol Optigrade^®^ for HPLC, Promochem, LGC Standards GmbH, Germany). The samples were then centrifuged at 17,000 × *g* for 10 min in a Microstar 17R centrifuge (VWR, United States), and the supernatants were transferred to standard HPLC vials.

Temperature and pH optimum were assessed by measuring Te formation with a 1260 Infinity II LC System (Agilent Technologies, United States). Samples were separated through an Agilent Poroshell 120 HPH-C18 column (Agilent Technologies, United States) with a gradient of acetonitrile 99.9% HPLC grade (Fischer Scientific, United States) and 0.1% (v/v) formic acid (98–100% Suprapur^®^, Sigma-Aldrich, United States) in Milli-Q water. The flow rate was set at 0.2 mL/min. One microliter of the sample was injected. Acetonitrile was increased from 5 to 44% until minute 12 and then to 70% at minute 15 remaining constant for other 3 min. MHET and Te formation was detected at 240 nm.

### Product Inhibition

The inhibition of Mle046 activity in the presence of the products Te (12,500, 10,000, 7,500, 5,000, and 2,500 μM) and 1,4-butanediol (B) (100,000, 200,000, and 450,000 μM) was tested. For this assay, the Te or B was diluted in 100 mM sodium phosphate buffer pH 7.5 in the presence of 1,000 μM of MpNPT (MpNPT was synthesized according to Palm et al., 2019) and 0.02 μg/μL of Mle046. Incubations were performed in triplicate at 20°C, in a Veriti^TM^ 96-Well Thermal cycler (Applied Biosystems, United States). Negative controls consisting of only the substrate and buffer were included together with positive controls consisting of the substrate, the buffer, and Mle046 without an inhibitory product.

The absorbance of the samples was measured after 30 min of incubation at 450 nm in a TECAN Infinite^®^ M200 plate reader (TECAN, Switzerland) and retrieved using the TECAN i-control v. 1.5.14.0 software (TECAN, Switzerland). The concentration of the produced 4-nitrophenol was obtained from a 4-nitrophenol standard curve prepared under the same assay conditions.

## Results

### Mle046 Purification and Identification

The Mle046 sequence obtained in a previous study (Meyer-Cifuentes et al., 2020) was used in this study and codon optimized for recombinant expression. The theoretical size of the His-tagged Mle046 is 62 kD. Structural analysis of the Mle046 sequence and its similarity to other MHETase-like enzymes were described in more detail previously (Meyer-Cifuentes et al., 2020).

**FIGURE 2:**
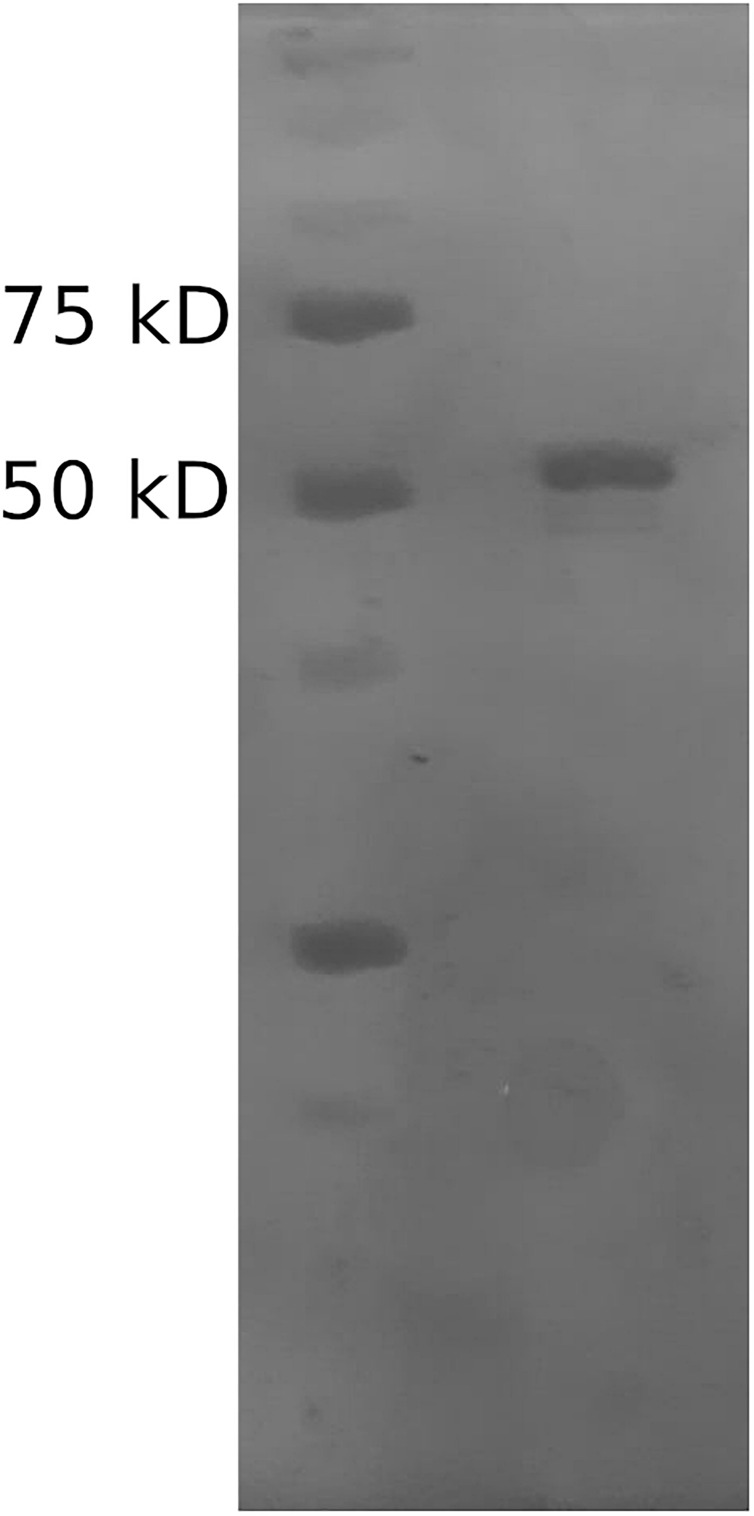
Purified His-tagged Mle046 protein. The size of the purified protein agrees with the predicted size (62 kD).

The Mle046 protein was purified by affinity chromatography to determine degradation kinetics, substrate specificity, inhibition, and optimized conditions. The size of the purified Mle046 on an SDS-PAGE gel was similar to the expected size. The purest fraction was used in this study (Figure 2). The concentration and calculated yield of Mle046 after purification were 4 mg/mL and 60 mg of protein per 1 L of cell culture, respectively.

### Mle046 Activity Toward MHET and Bte

**FIGURE 3:**
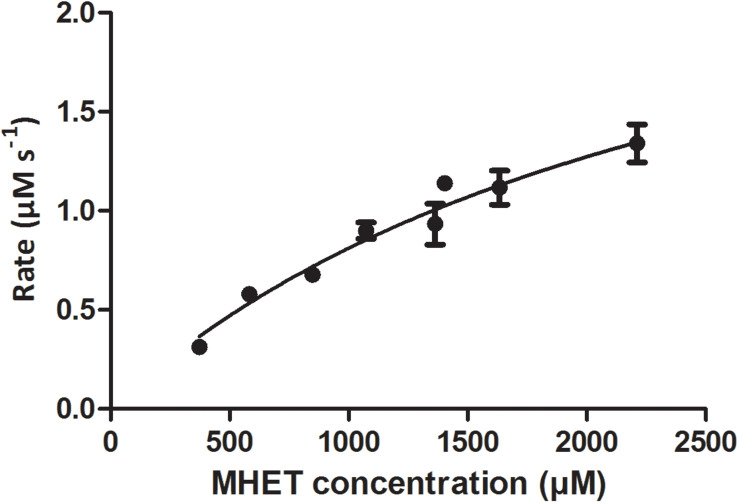
Rate of MHET catalysis by Mle046 plotted against substrate concentration. Degradation rates are expressed in terms of μM MHET degraded per second. Error bars indicate standard deviation (*n* = 3).

**FIGURE 4:**
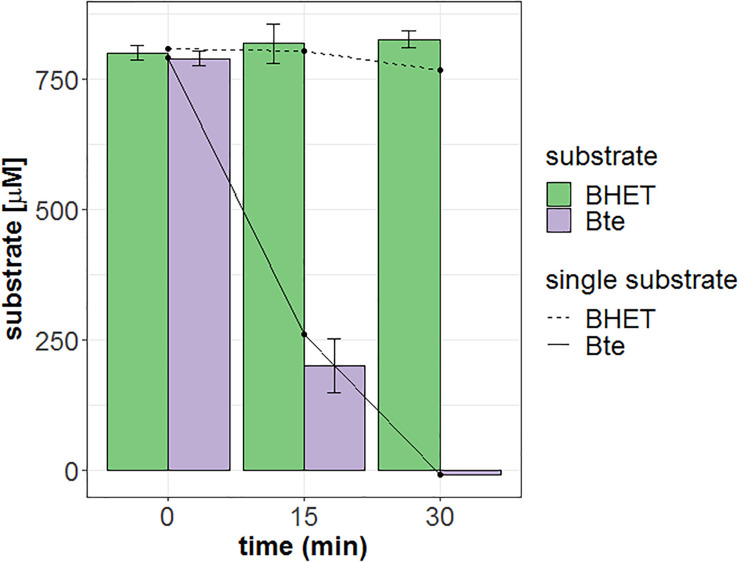
Degradation of Bte, BHET, or a mixture of both by Mle046. Bte and BHET supplied as single substrates are shown as solid and dashed lines, respectively. The mixture of Bte and BHET is shown as purple and green filled bars, respectively. The sampling points are shown at 0, 15, and 30 min. Error bars indicate standard deviation (*n* = 3).

Mle046 (0.002 μg/μL; 0.04 μM) can degrade MHET to concentrations up to 2,200 μM at 30°C. The highest rate was 1.4 μMs^–1^ with 1,570 μM of MHET. With 2,700 μM MHET, the rate of degradation declined to 0.47 μMs^–1^. As shown in Figure 3, Mle046 kinetics followed a Michaelis–Menten curve with concentrations up to 2,230 μM. Mle046 *Vmax*, *Km*, and the rate constant *kcat* were estimated as 2.9 μMs^–1^, 2,638 μM (±797), and 80.9 s^–1^ (±15.8), respectively (Supplementary Table 2). The reaction efficiency of Mle046, *kcat*/*Km*, was 0.03. After 21 min of incubation, Mle046 degraded 92% of 800 μM of MHET relative to the first sampling point. On the contrary, when 2,700 μM of MHET was present, the Mle046 could be degraded only ∼10% of MHET. We also tested the activity of Mle046 on the first intermediate of PF degradation, Bte. The incubation of Mle046 (0.002 μg/μL) in the presence of 1,000 μM Bte led to 94% Bte degradation after 21 min (Supplementary Figure 1). The degradation rate with 1,000 μM of Bte, 0.82 μMs^–1^, was almost the same as the degradation rate of Mle046 incubated with 1,140 μM MHET, 0.78 μMs^–1^. After 21 min of incubation with 1,000 μM of Bte with Mle046, 861 μM of Te was formed. When Mle046 (0.005 μg/μL) was incubated with 800 μM Bte, 800 μM of BHET, or a mixture of both substrates, the degradation rate of Bte remained the same (0.62 ± 0.03 μMs^–1^) with either of the substrates or in combination. Whether Bte was supplied alone or together with BHET, more than 70% of the initial amount of Bte was degraded after 15 min (Figure 4). This suggests that BHET has no inhibitory effect over Bte degradation. At the same time, Mle046 had no activity toward BHET.

### Dependence of Mle046 Activity on Temperature and pH

**FIGURE 5:**
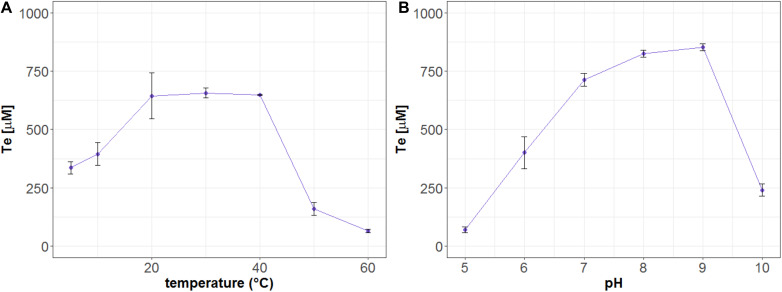
Formation of Te (μM) after hydrolysis of MHET by Mle046. The release of Te by Mle046 was quantified after 20 min at **(A)** different temperatures and **(B)** different pH values. Error bars indicate standard deviation (*n* = 3).

Mle046 degraded MHET and produced Te at every temperature tested. At 20, 30, and 40°C, after 20 min of incubation, Mle046 released similar amounts of Te 644, 656, and 648 μM, respectively (Figure 5A). The released amount of Te decreased relative to the amount formed at 30°C; to 51% at 5°C, 60% at 10°C, 24% at 50°C, and 10% at 60°C. Mle046 formed the same amount of Te (98%) at 20 and 40°C.

We also analyzed the effect of pH on MHET degradation by the Mle046. Similar to the Mle046 activity at different temperatures, MHET was degraded in a broad range of pH conditions. As observed in this study, the activity of Mle046 was mostly affected by highly acidic (pH 5) or alkaline (pH 10) conditions (Figure 5B). The highest amount of Te was formed at pH 8 and 9 (839 ± 15 μM). Relative to the condition at pH 8, Te formation decreased to 8% at pH 5 and less than 30% at pH 10. Thus, the optimum pH of the enzyme activity is within a range of 7–9.

### Mle046 Product Inhibition by Te and B

**FIGURE 6:**
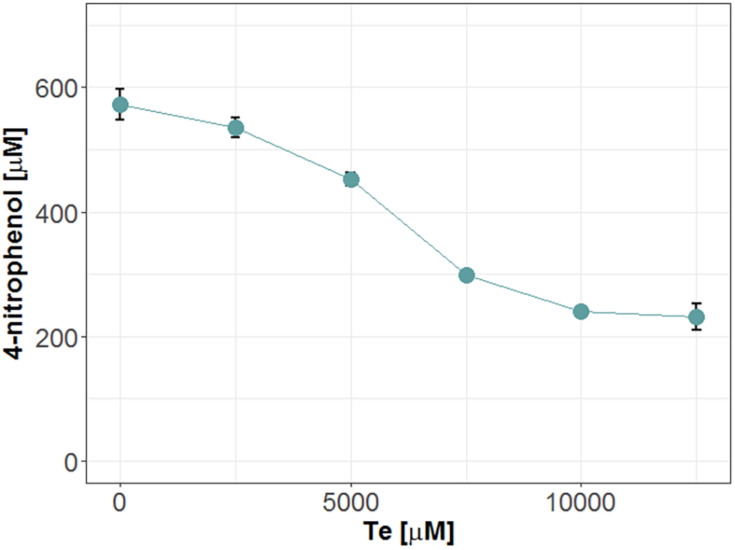
Release of 4-nitrophenol after hydrolysis of MpNPT by Mle046 and in the presence of various concentrations of Te. Incubations were carried out for 30 min. Error bars indicate standard deviation (*n* = 3).

To test product inhibition, we incubated Mle046 at 20°C in the co-presence of MpNPT and Bte degradation products: Te or B. The activity was then assessed by measuring the formation of 1,4-nitrophenol. Under these conditions, we observe that high concentrations of Te had a negative effect on the degradation of MpNPT by Mle046 (Figure 6). When we incubated Mle046 in the presence of Te concentrations of ≥7,500 μM, less than 50% of 4-nitrophenol was formed (relative to Mle046 without Te). With lower concentrations (<5,000 μM), Mle046 produced the same amount of 4-nitrophenol as when Mle046 was incubated in the absence of Te or B. In the presence of 2,500 μM of Te, Mle046 formed 530 μM of 4-nitrophenol similarly to the 570 μM 4-nitrophenol formed in the absence of Te. This means that with 2,500 μM of Te, the degradation rate was reduced only by 7%. Contrary to Te, none of the B concentrations tested in this study had a negative effect on the formation of 4-nitrophenol by Mle046 (data not shown).

## Discussion

In this study, we expressed, purified, and biochemically characterized an MHETase-like enzyme, Mle046, identified previously in a marine microbial consortium (Meyer-Cifuentes et al., 2020). To the best of our knowledge, this is the first identified and characterized marine MHETase-like enzyme.

We show that Mle046 degrades not only the first intermediate of PF degradation, Bte, but also the first intermediate of PET degradation, MHET. Mle046 can degrade MHET over a wide range of concentrations at 30°C. Its *Km* is, however, high (2,638 μM ± 797) when compared to those of IsMHETase (7.3 ± 0.6 μM) (Palm et al., 2019) and IsMHETase homologs of *Comamonas thiooxydans* and *Hydrogenophaga* sp. PML113 (174.70 ± 4.75 and 41.09 ± 3.38 μM, respectively) (Knott et al., 2020). This implies that Mle046 has a lower affinity to MHET and that the enzyme does not accept MHET as efficiently as the previously described homologs. However, enzymes which catalyze the same reaction but are from different organisms can have widely differing *Km* values (Robinson, 2015). As already suggested by Palm et al. (2019), low substrate affinity can be a disadvantage for bacteria in natural environments where low substrate concentrations are expected. The turnover rate (*kcat*) of MHET by Mle046 was, however, much higher than those that were reported for other MHETases. The turnover of the IsMHETase calculated in different studies ranges between 11.1 and 31 s^–1^ (Yoshida et al., 2016; Palm et al., 2019; Knott et al., 2020), while the turnover of *Hydrogenophaga* sp. PML113 and *C. thiooxydans* MHETases is 3.8 ± 2.5 and 9.5 ± 0.8 s^–1^, respectively (Knott et al., 2020). Due to the higher km value, the catalytic efficiency of Mle046 (0.03) is ∼40 times lower than that of IsMHETase but in the same order of magnitude as the *Hydrogenophaga* sp. PML113 and *C. thiooxydans* MHETases (Knott et al., 2020). Knott et al. (2020) demonstrated that an S131G mutant of the IsMHETase had a lower affinity to MHET and a much higher *Km* than the wild type. The position 131 is a glycine in the Mle046 (Gly117), as it is in the two homologs in *Hydrogenophaga* sp. PML113 and *C. thiooxydans* (Supplementary Figure 2). We therefore suggest that this residue is a determining factor for the substrate affinity of Mle046.

We also investigated if Mle046 is subjected to product inhibition, similar to IsMHETase, its homologs and other feruloic acid esterases (Crepin et al., 2003; Knott et al., 2020). We did not observe a strong inhibition of Mle046 by Te, one of the products of MHET and Bte degradation. Palm et al. (2019) proposed that substrate recognition by IsMHETase strongly relies on the aromatic ring of the substrate and that, therefore, Te might bind and inhibit the IsMHETase. Knott et al. (2020) suggested that the aforementioned S131G mutation reduces inhibition due to the reduced affinity of the enzyme to the substrate, which may explain the lack of Te inhibition for Mle046 as well. In the context of plastic degradation in the natural environment, Te inhibition would be less limiting, since this product is rapidly metabolized (Meyer-Cifuentes et al., 2020).

IsMHETase and some homologs such at TfCa from *Thermobifida fusca* present activity toward BHET (Belisário-Ferrari et al., 2019; Sagong et al., 2020). Yet, when Mle046 was incubated with Bte and BHET, Mle046 could not degrade BHET. In addition, the presence of this substrate did not inhibit Mle046 activity toward Bte. High specificity of Mle046 toward MHET might be beneficial in dual-enzyme systems designed to degrade plastics with a mixture of PETases and MHETases enzymes (Knott et al., 2020).

We also found that Mle046 is active at low temperatures, retaining ∼50% of its activity at 5°C compared to 30°C. We conclude that Mle046 is a mesophilic but cold-active enzyme, rather than a psychrophilic enzyme. At higher temperatures (>40°C), however, we observed a decline in Mle046 activity which indicates that Mle046 is heat-labile to a certain degree but not completely inactivated, retaining some of its activity at temperatures up to 60°C. The mesophilic and cold-active tendency of Mle046 is likely associated with the marine nature of the Mle046-producing bacteria were collected from. Mle046 originates from a mixed culture that was enriched using inocula collected from the North, Aegean, and Tyrrehenian seas. Overall, the surface temperatures of these areas range between 15 and 25°C yearly. Previously, we have shown that Mle046 was acquired via a transposable element from an Alphaproteobacterial conjugative plasmid, pDY25-B. Within this group, *C. manganoxidans* and *Sulfitobacter* sp. HI0023 contain the pDY25-B plasmid with the incorporated *mle046* sequence. *Celeribacter* species have been commonly isolated either from coastal surface seawater or deep-sea sediments (Lee et al., 2012; Baek et al., 2014; Lai et al., 2014; Wang et al., 2015). Like *Celeribacter* species, *Sulfitobacter* sp. HI0023 was isolated from the Pacific Ocean (Sosa et al., 2015). These findings strongly indicate that Mle046 contained in the pDY25-B plasmid evolved and adapted together with its hosts to mild-to-cold temperatures in the oceans.

Mle046 remains active at temperatures above 30°C and up to 60°C albeit at lower degradation rates. The activity of IsMHETase on the other hand rapidly falls at temperatures above 45°C and at 60°C the enzyme is virtually inactivate (Palm et al., 2019). Thus, Mle046 is an enzyme that can be used in two-enzyme degradation systems where high temperatures, typically around 60°C, are needed for more efficient PET degradation (Barth et al., 2015; De Castro et al., 2017; Wei and Zimmermann, 2017; Samak et al., 2020). In these systems, the PETases and MHETase-like enzymes can be additionally engineered to improve their hydrolytic properties (Austin et al., 2018; Ma et al., 2018; Son et al., 2019, 2020; Knott et al., 2020).

We conclude that Mle046 is a marine MHETase which can degrade the degradation intermediates of both PET and PBAT. With its activity at a broad range of temperatures and pH values, it is a contender as an enzyme to complement PETase activity in dual-enzyme systems for biotechnological applications. Engineering of the active site can improve the catalytic efficiency and substrate affinity.

## Data Availability Statement

The data presented in the study are deposited in the Genbank repository, accession number MZ408123.

## Author Contributions

IM-C designed and performed the experiments, analyzed the data, and wrote the manuscript. BÖ designed the experiments, analyzed the data, wrote the manuscript, and supervised the project. Both authors contributed to the editing of the manuscript and agreed on the final version.

## Conflict of Interest

The authors declare that the research was conducted in the absence of any commercial or financial relationships that could be construed as a potential conflict of interest.

## Publisher’s Note

All claims expressed in this article are solely those of the authors and do not necessarily represent those of their affiliated organizations, or those of the publisher, the editors and the reviewers. Any product that may be evaluated in this article, or claim that may be made by its manufacturer, is not guaranteed or endorsed by the publisher.
