# Supplementary material for: Mle046 Is a Marine Mesophilic MHETase-Like Enzyme
Source: Front Microbiol. 2021 Jul 26;12:693985. doi: 10.3389/fmicb.2021.693985 (PMC8351946; doi:10.3389/fmicb.2021.693985)
Supplement: Supplementary file 1 [file Data_Sheet_1.pdf]

Supplementary Material

1. Synthesis of Bte

Bte was synthesized according to the protocol from Perz et al. (Perz et al., 2016). Briefly, 23.1 g 1,4- butanediol (0.256 mol) and 12.4 g pyridine (0.157 mol) were combined and cooled to 0 °C. A solution of 6.13 g (0.03 mol) methyl 4-(chlorocarbonyl) benzoate (Sigma-Aldrich, USA) in 23.3 g of dichloromethane was added dropwise over 15 minutes. The reaction was stirred for 2 h at room temperature. The reaction was then poured over 200 mL water and brought to pH 1 with 150 mL of 1 M HCL. The aqueous phase was extracted three times with dichloromethane. The solvent was removed under reduced pressure to yield 6 g of methyl hydroxybutyl terephthalate. This product (6 g, 0.023 mol) was dissolved in 238 mL of a 1:1 mixture of methyl tert-butyl ether and chloroform. Dihydropyran (4.3 g, 0.06 mol) was added to the mixture dropwise over 15 minutes. The pH was adjusted to 4 with 0.22 g concentrated HCl. The reaction was stirred overnight at room temperature. It was then washed with 100 ml of saturated sodium bicarbonate solution and 100 ml water. The solvent was dried and removed under reduced pressure. Ten grams of crude product was purified by flash chromatography (hexane:ethyl acetate 8:1) to yield 5.3 g of the dihydropyranyl derivative of methyl hydroxybutyl terephthalate. 5.08 g of the dihydropyranyl derivative of methyl hydroxybutyl terephthalate and 2.67 g DABCO (23 mmol) were combined in a tube. The tube was sealed and heated to 100 °C for 4 h. The oil was taken up in 100 mL water at room temperature. Twenty five mL of 10% H_2SO_4 was added to this solution. The reaction was heated to 90°C, diluted with 75 mL of water and cooled to room temperature. After stirring at room temperature for a further two hours, the precipitate was filtered, washed twice with 20 mL of water and dried. Recrystallization from toluene yielded 2.1 g of monohydroxybutyl terephthalate. The structure was confirmed with ^{1}H NMR obtained on a Bruker Avance III platform (Bruker, USA). The purity was estimated to be 93% by ^{1}H NMR (500 MHz, DMSO-d6) δ 13.34 (s, 6H), 8.06 (s, 28H), 4.45 (s, 6H), 4.38 (s, 2H), 4.31 (t, J = 6.6 Hz, 13H), 3.89 (q, J = 1.0 Hz, 1H), 3.45 (d, J = 12.9 Hz, 13H), 3.31 (s, 3H), 1.93–1.87 (m, 1H), 1.82–1.67 (m, 13H), 1.61–1.50 (m, 13H) (Meyer-Cifuentes et al., 2020).

2. Supplementary Tables

Supplementary Table 1. Gene sequence of the codon optimized *mle046*.

Codon-optimized *mle046*	catatggcagttgcaggtgacttcccgaatcagttcctgagctgcttcgatgcagccaatctg<br>accgaaattgaactgccggcagatgttcagggcttccgtctgattgaaattgccgaacatgc<br>cggtgataaaggcatgccggcccattgtgaaattgtgggcgccattaatgatcgcattagtcc<br>ggtggatggccagcattatagcattaaattccgtctgcgtctgccgcaggattggaatggtcg<br>cttctatatggaaggcggtggcggtagcaatggtgttctgaaagatgcaatgggcccgacc<br>ggcctgaatcaggaagatagtgccctggaacgcggcttcgcagtggtgaccaccgatagc<br>ggccatgataatgataccaatagcgatagcaatgccagcggccgcagcgccttcggtatgg<br>atccgcaggcacgcctggacttcggttatatgagttatgatattgttacccgtgtgggtaaagc<br>aattgtggaaaaatattatggtgcagccccggaaaaaagttacttcattggttgtagtgaagg<br>cggtcgtgaagcagccctgatgacccagcgctatccggatctgtatgatggtgtggttgccg<br>gtgccccgggtattcacttcagctatagtgcagcctatgccccgttcctgctgcgcatcttcgg<br>taatctggccgaaaccagaaatcagagcggtccggatggtattccgctgctgaataaactgt<br>atagtgataatgatgtgcagctgattgccgatgccgttgtgggcgcctgtgatgccctggatg<br>gcctggaagatcgtatgagcaataatattgaagcctgtaccaccgttaccgtgctgccgcgc<br>ctgcgcgctctgacatgcagtggcgccaaagaagaaggttgcctgctggaagatcagattg<br>atgccttcgtggccggtatggcaggtccggtgaccagtgatggtacccgtctgtatccgggt<br>catccgtgggatccgggtattggcggtcgtattggtgatagtgttaatgatggcttccgtagtt<br>ggtggttcggtagctatgatagcgatcagaataatgcccgcaaagtgaccctgagcacccc<br>gcagcatgccatgctgtggcagaccccgccggttccgctgcgtcctgatgaatatgtgcgct<br>tcgaaatgaacttcaatattgatgaaacacctgccctggcatacgctaccaccgatctgtatcc<br>ggtgagcagtgcagaactgggcaatgccgatagtccggatctgagcgacttcgcaagccg<br>cggcggtaaactggtgatctatcatggtgccgcagatgcagcattcagtgcactggatacca<br>ttaaatattggaatgccgttaatgaaaccgccgatggtcaggccgcagacttcgcacgtctgt<br>tcattattccgggtatgaatcattgccagggtggtccggccaccgatgatgttgatctgctgac<br>cccgctgatggcatgggttgaagatgatctgccgattgaacgcctggaagccaccgtgagc<br>aatccggattacttcggtggtaaaaatctgagtcgcccgctgtgtccgtatccgctgtatgcc<br>gaatatgatggtgaaggtgatccgagtagcgcagaatcattcacctgtgtggccaataagctt

Supplementary Table 2. Kinetic parameters of Mle046. The kinetic parameters were determined in pH 7.5 buffer at 30°C. Parameters were calculated with GraphPad

Best-fit values	
Et	0.0365 µM
kcat	80,87 s^{-1} (±15,84)
Km	2,638 (±797)
Vmax	2.952 μMs^{-1}
95% CI (profile likelihood)	
kcat	47.92 to 113.8 s^{-1}
Km	980.8 to 4296
Goodness of Fit	
Degrees of Freedom	21
R squared	0.8960
Sum of Squares	0.2590
Sy.x	0.1110
Number of points analyzed	23

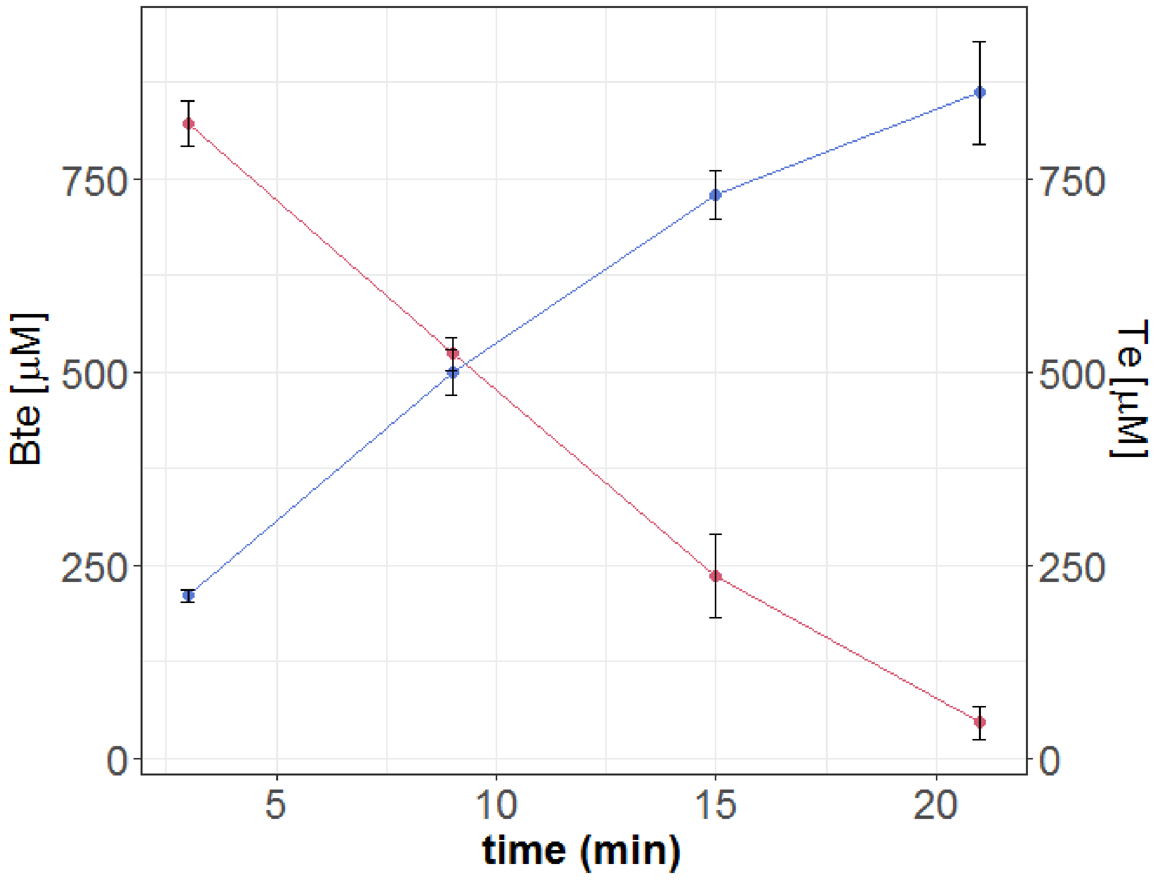

Supplementary figure S1. Bte degradation by Mle046. Bte degradation by Mle046 is shown in red on the left y-axis and Te formation in blue on the right y-axis. Four sampling points are shown: 3, 9, 15 and 21 min. Error bars indicate standard deviation (n=3).

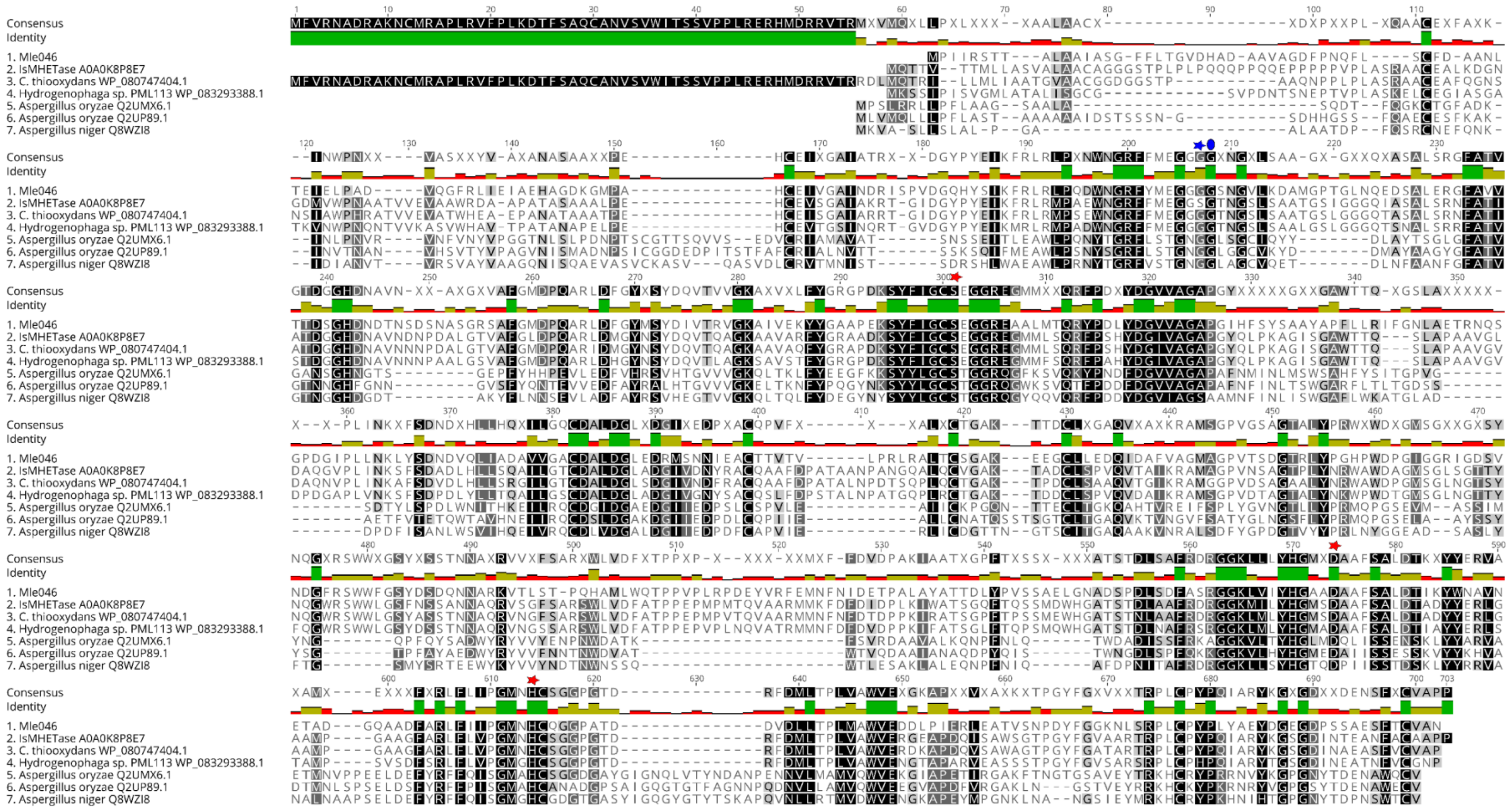
Consensus
Identity
1. Mle046
2. IsMHETase A0A0K8P8E7
3. C. thiooxydans WP_080747404.1
4. Hydrogenophaga sp. PML113 WP_083293388.1
5. Aspergillus oryzae Q2UMX6.1
6. Aspergillus oryzae Q2UP89.1
7. Aspergillus niger Q8WZI8

Supplementary figure S2. Pairwise alignment of Mle046 to MHETase homologs. Pairwise alignment of Mle046 homologues was performed with Clustal Omega. The NCBI/UniProt accession number for each homolog is given. The blue circle depicts the oxyanion hole. The red stars depict the catalytic triad. The blue star is the position S131 in IsMHETase.

References

Meyer-Cifuentes, I.E., Werner, J., Jehmlich, N., Will, S.E., Neumann-Schaal, M., and Öztürk, B. (2020). Synergistic biodegradation of aromatic-aliphatic copolyester plastic by a marine microbial consortium. *Nat. Commun.* 11**,** 5790.doi: 10.1038/s41467-020-19583-2

Perz, V., Bleymaier, K., Sinkel, C., Kueper, U., Bonnekessel, M., Ribitsch, D., et al. (2016). Substrate specificities of cutinases on aliphatic–aromatic polyesters and on their model substrates. *New Biotechnol.* 33**,** 295-304.doi: 10.1016/j.nbt.2015.11.004
